# Tumor-Derived Small Extracellular Vesicles Involved in Breast Cancer Progression and Drug Resistance

**DOI:** 10.3390/ijms232315236

**Published:** 2022-12-03

**Authors:** Lingyun Feng, Lijuan Guo, Yoshimasa Tanaka, Li Su

**Affiliations:** 1 Key Laboratory of Molecular Biophysics of Ministry of Education, College of Life Science and Technology, Huazhong University of Science and Technology, Wuhan 430074, China; 2 Center for Medical Innovation, Nagasaki University, 1-7-1, Sakamoto, Nagasaki 852-8588, Japan

**Keywords:** small extracellular vesicle, biomarkers, tumor microenvironment, breast cancer

## Abstract

Breast cancer is one of the most serious and terrifying threats to the health of women. Recent studies have demonstrated that interaction among cancer cells themselves and those with other cells, including immune cells, in a tumor microenvironment potentially and intrinsically regulate and determine cancer progression and metastasis. Small extracellular vesicles (sEVs), a type of lipid-bilayer particles derived from cells, with a size of less than 200 nm, are recognized as one form of important mediators in cell-to-cell communication. sEVs can transport a variety of bioactive substances, including proteins, RNAs, and lipids. Accumulating evidence has revealed that sEVs play a crucial role in cancer development and progression, with a significant impact on proliferation, invasion, and metastasis. In addition, sEVs systematically coordinate physiological and pathological processes, such as coagulation, vascular leakage, and stromal cell reprogramming, to bring about premetastatic niche formation and to determine metastatic organ tropism. There are a variety of oncogenic factors in tumor-derived sEVs that mediate cellular communication between local stromal cells and distal microenvironment, both of which are important in cancer progression and metastasis. Tumor-derived sEVs contain substances that are similar to parental tumor cells, and as such, sEVs could be biomarkers in cancer progression and potential therapeutic targets, particularly for predicting and preventing future metastatic development. Here, we review the mechanisms underlying the regulation by tumor-derived sEVs on cancer development and progression, including proliferation, metastasis, drug resistance, and immunosuppression, which coordinately shape the pro-metastatic microenvironment. In addition, we describe the application of sEVs to the development of cancer biomarkers and potential therapeutic modalities and discuss how they can be engineered and translated into clinical practice.

## 1. Introduction

According to the World Health Organization, there were 2.3 million women diagnosed with breast cancer (BC) and 685,000 deaths globally in 2020. Breast cancer typically arises in the lining cells of the ducts or lobules in the glandular tissue of the breast. BC cells exhibit distinct traits, such as high proliferation, self-renewal potential, cancer stem cell characteristics, metastasis, and the ability to switch between multiple molecular pathways to acquire drug resistance [1,2]. Eukaryotic cells secrete vesicles with membranous structures, which impact on both peripheral and distal cells [3]. Small extracellular vesicles (sEVs) are non-replicable lipid bilayer particles secreted into extracellular spaces by prokaryotic and eukaryotic cells with a size of < 200 nm [4,5,6]; they were once considered to be cells or solid wastes produced upon cell injury. However, subsequent studies revealed that sEVs play critical biological roles in the cellular microenvironment. Raposo et al. identified a variety proteins, lipids and nucleic acids in sEVs [7,8]. Cell-to-cell communication is achieved through the transfer of a plethora of bioactive molecules among cells via sEVs [9]. sEVs have been isolated from a variety of body fluids and shown to play unique roles not only in normal physiological functions, but also in pathologic processes, including angiogenesis, immune suppression, tumor metastasis, and chemoresistance [9,10,11]. sEVs separation methods include traditional high-throughput techniques, such as differential ultracentrifugation, density gradient centrifugation, precipitation, filtration, and size exclusion chromatography, as well as novel methods, including microfluidics and immune isolation [12]. Mechanisms underlying the internalization of sEVs into target cells have been elucidated. sEVs bind to cell surface receptors, such as integrins, tetraspanin transmembrane proteins, and intercellular adhesion molecules, and are internalized into cells via clathrin-mediated endocytosis, caveolae/lipid raft-mediated endocytosis, clathrin- and caveolin-independent endocytosis, micropinocytosis, and phagocytosis as the main pathways [13,14,15,16,17].

Recent studies suggest that sEVs could be used as cancer biomarkers and as potential targets for tissue regeneration, as well as cancer treatment. For instance, sEVs secreted by mesenchymal stromal cells have been employed to induce tissue regeneration after myocardial infarction [18,19,20], and dendritic cell (DC)-derived sEVs for cancer immunotherapy [21]. Furthermore, sEVs are used to load miRNAs and siRNAs for therapeutic applications due to their low immunogenicity and side effects. sEVs could, therefore, be potential drug carriers [22,23].

This review summarizes physiological and pathological aspects of sEVs, emphasizing their roles in cancer development and exploring their significance in tumor response prediction and application to cancer treatment.

## 2. Tumor-Derived sEVs in Breast Cancer

### 2.1. Characterization of Tumor-Derived sEVs in Breast Cancer

It is difficult to precisely define and classify the types of EVs of interest, because pre-secreted EVs are modified through multiple biogenetic pathways. The International Society for Extracellular Vesicles (ISEV) has, thus, issued a guideline for the characterization of EVs and the determination of the purity of EVs preparations. ISEV 2018 recommends that researchers employ nomenclature to specify physical characteristics of EVs, such as size (sEVs may be designated as <200 nm and medium/large EV > 200 nm), density, presence of certain biochemical components (e.g., CD63^+^ EVs), origins, and conditions of isolation (e.g., brain-derived EVs). The use of inducers and secretory inhibitors in sEVs isolation has to be stated to clarify putative sEVs characteristics or to distinguish sEVs from non-sEVs products. Exosomes (50–150 nm), microvesicles (100–1000 nm), and apoptotic bodies (1000–5000 nm) are classified as EVs based on particle size and generation mechanism [11,12,24]. Exosomes are released through exocytosis of multivesicular bodies via the Rab27a/b pathway, whereas microvesicles and apoptotic bodies are released via plasma membrane blebbing [25,26]. The composition of the sEVs’ surface molecules and the condition of the recipient cells dictate the route of uptake and the fate of the sEVs cargo [27]. sEVs are secreted into the extracellular space by cancer cells and constitute a part of tumor microenvironment, where a vast variety of bioactive molecules interact with the immune system [28,29]. Large volumes of data on protein types and abundance in sEVs are available and stored in public EV online databases, such as Vesiclepedia, EVpedia and ExoCarta [30,31,32].

Here, we discuss the functions of sEVs in breast cancer (BC) proliferation, metastasis, anti-cancer drug resistance and therapy. However, their own intrinsic functions in cancer remain elusive because most current purification techniques and functional studies fail to distinguish exosomes from plasma membrane-derived vesicles.

### 2.2. Tumor-Derived sEVs Facilitate Tumor Development

Tumor-derived sEVs participate in intercellular communication by transporting various molecules, including proteins, RNAs, and DNAs, which exhibit an oncogenic effect on cancer. Emerging evidence has suggested that tumor-derived sEVs could promote tumor growth and metastasis [33,34]. Li et al. demonstrated that tumor-derived sEVs containing c-Myc activated KCNQ1OT1, down-regulated miR-556-3p, and subsequently increased CLIC1 expression to activate the PI3K/AKT pathway, promoting gastric cancer growth and metastasis [33]. Ruivo et al. showed that cancer stem cell-derived sEVs armed with agrin protein can activate Yes1-associated transcriptional regulator (YAP) via low-density lipoprotein receptor-related protein 4 (LRP-4). In-vitro anti-agrin treatment of PDX significantly inhibited proliferation and decreased the level of activated YAP. The disease-free survival of patients with high levels of agrin and low levels of activated YAP was poor [34]. 

### 2.3. Tumor-Derived sEVs Promote Cancer Metastasis

sEVs are hypothesized to be involved in numerous processes during the invasion of cancer cells and may contribute to the early stages of metastasis (Table 1). Tumor cells can exchange and share oncogenic molecules through sEVs. Tumor metastasis is a multistep process, in which locally advanced tumor cells disseminate into distant organs. Tumor-derived sEVs are involved in the alteration of a variety of metastatic pathways, responsible for tumor invasion [35,36]. sEVs, for example, play an essential role in the formation of invasive pseudopods, critical for the invasion and metastasis [37,38]. Tumor-derived sEVs modulate tumor cells by altering the integrity of the vascular barrier. Melanoma-secreted sEVs have been shown to cause blood vessel leakage in the lungs [39]. Proteomics analysis of tumor-associated sEVs revealed that sEVs release proteins, including SERPINA1, SERPINF2, and matrix metalloproteinase 9 (MMP9), which are involved in extra cellular matrix remodeling, vascular leakage, and aggressiveness [40]. In vitro, glioblastoma cell-derived sEVs with high levels of vascular endothelial growth factor (VEGF)-A increase endothelial cell permeability and angiogenesis [41]. In addition, sEVs secreted from lung cancer and BC cells carry miR-23a and miR-105, respectively, both of which target ZO-1 protein, increasing vascular permeability and, thereby, boosting cancer cell trans-endothelial migration [42,43]. Rab27b mediates the release of HSP90-containing sEVs to activate MMP2 in metastatic BC cells, resulting in TME component breakdown, growth factor release, and promotion of cancer cell invasion [44]. Tumor-derived sEVs carrying miR-222 promote BC cell migration and invasion by downregulating the tumor suppressor PDLIM2 and activating the NF-κB signaling pathway [45]. Hoshino et al. reported that sEVs mediated organ tropism and premetastatic niche generation by secreting different integrins (e.g., integrin α6 β4, α6 β1, or α5). Integrins α4 and α6 β1 were inexorably linked to lung metastasis, while integrin αvβ5 was associated with liver metastasis [46]. Notably, cancer cells secrete sEVs carrying high quantities of miR-122, which reprograms stromal cells towards glucose metabolism by targeting pyruvate kinases. This, in turn, increases nutrients in the pre-metastatic niche, facilitating metastasis [36]. These data demonstrate that sEVs play a crucial role in regulating metastatic niches through various proteins and microRNAs.

It is worthy of note that chemotherapy has pro-metastatic effects in animal models. Keklikoglou et al. demonstrated that taxanes and anthracyclines, two types of cytotoxic drugs commonly used in pre-operative (neoadjuvant) BC treatment, promoted secretion of sEVs with pro-metastatic ability. Chemotherapy-induced sEVs contains a high level of annexin A6 (ANXA6), a Ca^2+^-dependent protein, that stimulates NF-κB-dependent endothelial cell activation, C-C motif chemokine 2 (CCL2) induction, and Ly6c^+^/C-C-chemokine receptor 2 (CCR2^+^) monocyte proliferation in the lung pre-metastatic niche to facilitate lung metastasis [47].

**Table 1:** Mechanisms underlying tumor growth and metastasis mediated by BC-derived sEVs.

Molecules in sEVs	Target Cells	Functions	Ref.
TSP1	Endothelial cells	TSP1-enriched exosomes promoted the transendothelial migration of malignant cells and decreased the expression of intercellular junction proteins. Exosomal TSP1 facilitated the transendothelial migration of breast cancer cells via disrupting the intercellular integrity of endothelial cells.	[48]
miR-105	Endothelial cells	Cancer-secreted miR-105 efficiently destroys tight junctions and the integrity of these natural barriers against metastasis.	[42]
CEMIP	Endothelial cells	Promotion of vascular remodeling, leading to the formation of a pre-metastatic niche.	[49]
TGF-β	Fibroblast, bone marrow myeloid cells	Transducing SMAD-dependent signaling. Inhibiting either signaling or β-glycan expression attenuated differentiation.	[50,51]
Survivin	Fibroblasts	Breast cancer cell-derived Survivin upregulates SOD1 expression in fibroblasts and converts them into myofibroblasts, conversely inducing breast cancer progression in vitro and in vivo.	[52]
miR-9	Fibroblasts	Enhancing the switch to CAF phenotype, thus contributing to tumor growth.	[53]
Myosin-9	Macrophages	SIPA1-high breast cancer can enhance macrophage infiltration through sEVs enriched with myosin-9, which might aggravate the malignancy of breast cancer.	[54]
gp130	Macrophages	Breast cancer cell-derived exosomes transfer gp130 to macrophages in vitro, which alters macrophage polarization by activating the STAT3 pathway.	[55]
EGFR	Macrophages	Breast cancer cell-derived exosomes transferred activated EGFR to host macrophages, which inhibited their production of type I interferons and antiviral immunity, resulting in compromised innate immunity.	[56]
PGE2	Bone marrow myeloid cells	MDSC-mediated promotion of tumor progression is dependent on T-exosome prostaglandin E2 (PGE2) and TGF-β molecules. T-exosomes can induce the accumulation of MDSCs expressing COX-2, IL-6, VEGF, and arginase-1.	[51]
lncRNA SNHG16	Tregs	BC-derived exosomal SNHG16/miR-16-5p/SMAD5-regulatory axis potentiates TGF-β1/SMAD5 pathway activation, thus inducing CD73 expression in Vδ1 T cells.	[57]
TβRII	CD8 T cells	Malignant breast cancer cells can transfer active TGF-β type II receptor (TβRII) via tumor-derived extracellular vesicles (TEV) and, thereby, stimulate TGF-β signaling in recipient cells. EV-TβRII delivered as a cargo to CD8^+^ T cells induces the activation of SMAD3, which is associated and cooperated with TCF1 transcription factor to impose CD8^+^ T cell exhaustion.	[58]
Integrins α6β4 and α6β1		Associated with lung metastasis.	[46]
Integrin αvβ5		Linked to liver metastasis.	[46]
Palmitoylated proteins	Macrophages	NF-κB activation.	[59]
miR-122	Stromal cells	By preventing glucose uptake in stromal cells via miR-122-mediated inhibition of pyruvate kinase, breast cancer cells create a PMN with greater glucose availability for their own utilization.	[36]

### 2.4. Biomarkers of sEVs

Recent evidence shows that some sEVs proteins, miRNAs and LncRNAs have been upregulated in BC patients’ sera, suggesting that these sEVs could be diagnostic indicators for BC. Because only routine blood collection is required, sEV-based diagnostics is noninvasive and feasible. Chen et al. demonstrated that phosphoproteins in plasma sEVs could be disease biomarkers and this diagnostic strategy may facilitate cancer screening and surveillance [60].

Notably, a study that used plasma sEVs microRNAs as diagnostic biomarkers for BC patients indicated that these molecules enable us to distinguish between BC patients and noncancerous women. Zhai et al. successfully detected microRNA-1246 in situ at sEVs’ locations by harnessing a nucleic acid-functionalized Au nanoflare probe that infiltrated into plasma sEVs directly and generated fluorescence signals quantitatively [61]. 

The in-situ detection of the miRNA-1246 in sEVs in the peripheral blood could discriminate 46 BC patients from 28 healthy subjects with 100% sensitivity and 93% specificity at the appropriate cut-off point [61]. Another clinical study of sEVs microRNA signatured in 20 healthy women and 435 BC patients demonstrated that 10 miRNAs in the entire BC patient cohort, 13 miRNAs in the HER2^+^ subgroup, and 17 miRNAs in the triple-negative subgroup were significantly deregulated, compared to those in healthy women, indicating different aspects of cancer biology in different BC types [62].

**Figure 1:**
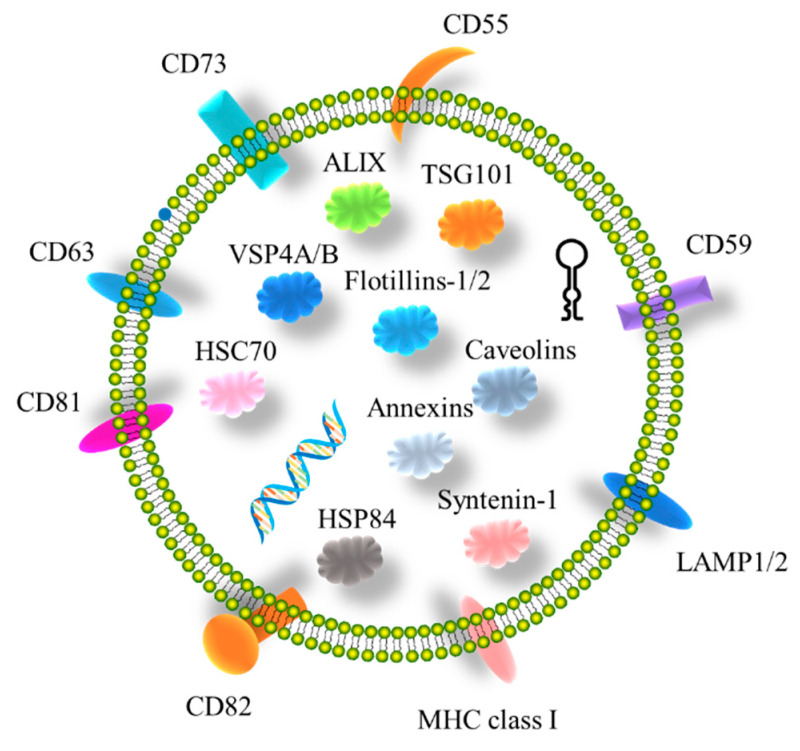
Biomarkers of sEVs. Transmembrane/lipid-bound proteins: CD55, CD59, CD63, CD73, CD81, CD82, LAMP1/2, and MHC class I; Cytosolic proteins: ALIX, Annexins, Caveolins, Flotillins-1/2, HSC70, HSP84, Syntenin-1, TSG101, and VSP4A/B.

The plasma membrane, cytosol, Golgi and nucleus proteins are most frequently detected in sEVs [63,64]. As more sEVs’ components were comprehensively listed, it became evident that sEVs contain both proteins and cell-type-specific molecules. Cytoplasmic membrane proteins, ribosomal proteins, heat-shock proteins, cytoskeletal proteins, annexins, tetraspanins, major histocompatibility complex (MHC), metabolic enzymes and vesicle trafficking-related proteins are frequently detected in sEVs, while organelle proteins are rare [65]. It was reported that four transmembrane proteins (CD9, CD63, CD81, and CD82), MHC proteins and cytoplasmic proteins (HSPs, TSG101, and Alix) were highly expressed in sEVs and were selected as biomarker candidates, in which CD9, CD63, and CD81 are frequently utilized as EV biomarker proteins (Figure 1), whereas a recent study indicated that they are also present in microvesicles and apoptotic bodies [66]. 

Comprehensive methodology and experimental conditions for sEVs isolation are, therefore, important to identify important sEVs markers. ESCRT-dependent routes can be used to perform MVB membrane budding and ILV cargo sorting [67,68]. Syntenin protein, which is highly and stably expressed in sEVs secreted by multiple cells, is required for ILV production [69,70]. Triple-negative BC (TNBC)-derived sEVs harbor UCHL1 (ubiquitin carboxyl-terminal hydrolase isozyme L1), which has been considered as a biomarker for TNBC [71]. Surface membrane proteins (e.g., CD151) found on TNBC-derived sEVs can be also used as biomarkers to diagnose TNBC [72]. The epidermal growth factor receptor (EGFR) and Survivin are two other examples of potential protein biomarkers for TNBC-derived sEVs [73,74,75]. Glutathione S-transferase P1 (GSTP1) expression was higher in the progressive disease (PD)/stable disease (SD) group than in the partial response (PR)/complete response (CR) group in both pre-and post-treatment samples. The levels of GSTP1 in serum exosomes from 30 patients having anthracycline/taxane-based neoadjuvant chemotherapy were significantly higher in the PD/SD group than those in the PR/CR group [76]. According to ClinicalTrials.gov, three studies (NCT04653740, NCT04258735 and NCT04530890) are currently ongoing to determine whether exosomes could be prognostic and reliable biomarkers in BC.

## 3. Tumor-Derived sEVs Play Vital Roles in the BC Microenvironment

### 3.1. Effects of Tumor-Derived sEVs on Angiogenesis

sEVs secreted by various cells in TME are key mediators of pathological angiogenesis that support tumor growth by packaging angiogenic factors transported to endothelial cells, through which a niche for angiogenesis is formed [77]. Tumor-derived sEVs promote angiogenesis in TME by releasing non-coding RNAs. miR-9 in tumor-derived sEVs, for example, initiates endothelial angiogenesis and migration by decreasing SOCS5 expression levels and activating the JAK-STAT signaling pathway in endothelial cells [78]. It has been shown that miR-23a-carrying sEVs induce angiogenesis in various angiogenic model systems by targeting SIRT1 in recipient endothelial cells [79]. Similarly, neutral sphingomyelinase 2 (nSMase2) promotes endothelial cell angiogenesis by transferring pro-angiogenic sEVs containing miR-210 [80].

Elevated heparinase levels in myeloma and BC cells have also been linked to increased release of syndecan-1, VEGF and hepatocyte growth factor (HGF) in sEVs, resulting in increased endothelial invasion via TME [81]. Tumor-derived sEVs contain a variety of angiogenic factors, including VEGF, fibroblast growth factor (FGF), platelet-derived growth factor (PDGF), basic fibroblast growth factor (bFGF), transforming growth factor-β (TGF-β), tumor necrosis factor-α (TNF-α), and IL-8 [82,83]. sEVs produced from human BC cells contain the ITGα6β4 protein, which acts on lung fibroblasts and Kupffer cells, activates the intracellular Src kinase signaling pathway, upregulates the S100 protein subset, and plays a role in lung metastasis of cancer cells by promoting vascular leakage [46]. The upregulation of heparinase in myeloma and BC cells is associated with increased release of sEVs containing syndcan-1, VEGF and HGF, and with enhanced tumor cell proliferation and massive invasion of endothelial cells through TME [81]. sEVs secreted from MDA-MB-231 BC cells contain the TSP1 protein, which breaks intercellular connections between endothelial cells and promotes tumor cell migration [48]. Annexin II (ANXA2), a tumorigenic factor, was recently discovered in sEVs secreted by BC cells. Angiogenesis was induced in vitro and in vivo through tPA-dependent mechanisms. The ANXA2 protein in sEVs has been proposed as a biomarker for the detection and targeted treatment of metastatic breast tumors [84].

### 3.2. Effects of Tumor-Derived sEVs on Fibroblast and Stromal Activation

Cancer-associated fibroblasts (CAFs) are one of the important components of tumor stroma. CAFs produce chemokines, cytokines and TME proteins, all of which are required for tumor architecture, growth, invasion and metastasis [85,86,87]. CAFs’ morphology and gene expression profiles are similar to those of myofibroblasts [86]. In response to PDGF and TGF-β signaling, myofibroblasts and CAFs can be differentiated from tissue fibroblasts. When normal fibroblasts differentiate into CAFs or myofibroblasts, they express α smooth muscle actin (αSMA) and caveolin-1 (CAV1) and secrete abundant MMPs, as well as multiple growth factors and cytokines including TGF-β, FGF2, HGF and CXCL12 [88,89,90]. Compelling evidence indicates that CAFs contribute to cancer progression; for example, tumor cells implanted together with CAFs show enhanced tumor growth and metastasis when compared to those with normal fibroblasts [91,92,93]. In addition, CAFs promote tumor cell invasion at the tumor margin via chemokines and cytokines [85]. In a nutshell, CAFs are required for cancer progression. When resident fibroblasts uptake human BC cells-derived sEVs containing miR-125b, the intracellular miR-125b was increased and the expression of numerous CAFs markers was markedly upregulated in the fibroblasts. In mouse and human fibroblasts, overexpression of miR-125b leads to an activated phenotype, which is comparable to that induced by the established miR-125b-targeted mRNAs. These findings imply that miR-125b is transferred from BC cells to normal fibroblasts within TME via sEVs and transforms them into CAFs [94].

Tumor-derived sEVs also promote the differentiation of MSCs and other bone marrow-derived cells into tumor-supportive cells by releasing growth factors, including TGF-β and miRNAs [95,96]. BC cells, for example, can convert cancer cells into normal fibroblasts and epithelial cells through tumor-derived sEVs containing the cross-linking enzyme tissue transglutaminase-crosslinked fibronectin [97]. It was also demonstrated that BC-derived sEVs enhanced the development of myofibroblast-like features in adipose-derived MSCs [98].

The transmission of non-coding oncogenic miRNAs is another mechanism for tumor-stromal remodeling via sEVs. Indeed, miR-9 in BC-derived sEVs induces the transition of human breast fibroblasts to CAFs and enhances cell motility [53]. Fibroblasts secrete sEVs via autocrine Wnt-planar cell polarity signaling, which promotes protrusive activity, motility and metastasis in BC cells [99]. In another study, BC cells treated with CAF-derived sEVs containing miRs-21, -378e, and -143 exhibited stemness and epithelial–mesenchymal transition (EMT) phenotypes [100].

### 3.3. Effects of Tumor-Derived sEVs on Immune Cells

Although anti-tumoral immune cells infiltrate into TME, tumor-derived sEVs suppress the immune system and allow tumor cells to escape from the immune attack. The induction of the immuno-suppressive environment is a critical process in cancer development. sEVs have been shown to be involved in the exhaustion of cytotoxic T-cells, the growth of regulatory T-cells, the polarization of macrophages to the M2 phenotype, the inhibition of cytotoxic natural killer (NK) cells, the immunosuppression of myeloid-derived suppressor cells (MDSCs) and the polarization of neutrophils to the N2 phenotype [57,101,102,103,104,105,106]. 

Tumor-associated macrophages (TAMs) promote cancer cell invasion and metastasis, leading to tumor progression [107]. TAMs are derived from circulating monocytes that are recruited into tumors via the CCL2-CCR2 chemokine signaling pathway [108,109]. The fate of monocyte is primarily regulated, rather than predefined, by the microenvironmental signals they encounter [110]. Cytokines and chemokines are reliable signature factors in the TAM differentiation and activation signaling [111]; in addition to the soluble factors, sEVs have been shown to be candidates as the novel immuno-suppressive factors. sEVs are complex intercellular communication vehicles that have been shown to regulate macrophage activation [59,112]. For instance, Feng et al. demonstrated that the myosin-9 protein in BC cells-derived sEVs could promote macrophage infiltration [54]. sEVs interact with recipient cells and modulate their activity once being released [113]. In particular, sEVs produced by tumor cells play a crucial role in shaping tumor immune microenvironment. Tumor cells-derived sEVs alter activity and function of lymphocytes and myeloid cells by eliciting protumor and anti-tumor immune responses [114,115], which is pegged on a range of parameters, such as cancer types, stages, and sEVs subtypes [116]. The combination of surface colony-stimulating factor-1 (CSF-1) promoting survival and cargoes promoting cyclic guanosine 5′-monophosphate (GMP)-adenosine monophosphate (AMP) synthase/stimulator of interferon genes (cGAS/STING) or other activation pathways in TNBC sEVs induces the differentiation of specific macrophage subsets. Notably, macrophages expressing sEV-induced signature have been observed in TAMs in patients. Furthermore, higher levels of this signature were correlated with T cell infiltration and prolonged patient survival. These findings suggest that TNBC-released CSF-1-bearing sEVs shape the tumor immune microenvironment and improve prognosis in TNBC patients [117]. In addition, sEVs secreted by BC cells subjected to chemotherapy increased lung metastasis, possibly due to the mobilization of immune cells supporting the pre-metastatic niches [47]. BC cells-derived sEVs also suppress anti-cancer immune responses in pre-metastatic organs by directly inhibiting T-cell proliferation and NK cell cytotoxicity, hence altering TME [118].

Wieckowski et al. demonstrated that tumor-derived sEVs carry Fas-L protein, which contributes to TME immune suppression by promoting Treg proliferation and CD8^+^ T cell apoptosis [119,120]. HSP72 and HSP105 in sEVs in the sera of melanoma, lung cancer and BC patients activate dendritic cells and trigger IL-6 production, which promote tumor invasion by increasing MMP9 expression [121]. MICA, a protein that binds to the NK cell receptor NKG2D, was found in tumor-derived sEVs, leading to suppression of NK cytotoxicity [122].

In a study analyzing human serum samples, miRNA-1246 and miRNA-21 levels were significantly higher in sEVs of BC patients compared to those of healthy individuals [123]. sEVs containing the MHC-peptide complex activated homologous T-cell receptors on T cells in immune responses [124]. Tumor-derived sEVs expressing PD-L1 were found on the surface of metastatic melanoma. PD-L1-sEVs attach to T cells and suppress T cell functions through PD-1/PD-L1 interaction. Breast and lung tumors also release PD-L1-sEVs [29]. In addition, macrophages in the lungs and brain uptake sEVs secreted by BC cells, leading to the activation of NF-κB signaling pathway and the secretion of pro-inflammatory cytokines IL-6, TNF-α, granulocyte colony-stimulating factor and CCL2 [59].

### 3.4. Effects of Microenvironment-Derived sEVs on BC Development

TME-derived sEVs are also crucial for the development and progression of BC. TME consists of stromal cells, such as fibroblasts, mesenchymal stromal cells, pericytes and adipocytes, and immune cells, including T and B lymphocytes, NK cells, and TAMs, all of which are embedded in the ECM [125,126]. Of note, CAFs are one of the major multiple stromal cell components of TME in breast, colon, pancreatic and prostate cancers [127,128,129]. CAFs exhibit highly heterogeneous cell types; some of which show anti-cancer activity, while others have a pro-oncogenic role [130]. For instance, CAFs in estrogen receptor (ER)-positive BC can be divided into two functional sub-groups with opposing roles based on CD146 expression. CD146-positive CAFs retain ER expression in ER-positive BC cells, as well as estrogen responsiveness and tamoxifen sensitivity, whereas CD146-negative CAFs inhibit cancer cell responses to tamoxifen, resulting in poor prognosis [131]. TGF-β secreted by CAFs induces EMT of BC cells via TGF-β/SMAD and non-SMAD signaling pathways [132,133]. Several miRNAs originating from CAF-secreted sEVs have been shown to play key roles in cancer progression. MiR-181d-5p, miR-500a-5p, miR-21, miR-22, miR-378e and miR-143, for instance, were increased in breast CAFs-derived sEVs, compared to those secreted by normal fibroblasts [100,134,135,136]. In BC, CAFs-derived sEVs containing miR-181d-5p promote cancer cell proliferation, invasion, migration and EMT while inhibiting apoptosis by targeting caudal-related homeobox 2 (CDX2) and its downstream gene-homeobox A5 (HOXA5) [134]. sEVs containing miR-500a-5p enhance proliferation and metastasis in BC cells by decreasing the expression of ubiquitin-specific peptidase 28 (USP28) [135]. Some circular RNAs (circRNAs) also play tumor-promoting roles in CAFs-derived sEVs. In BC, sEVs derived from hypoxic CAFs show higher circHIF1A levels than normoxic CAFs. circHIF1A acts as a sponge for miR-580-5p, lowering its levels, whilst miR-580-5p targets the CD44 molecule mRNA, decreasing CD44 expression [137]. Protein transfer from CAFs to cancer cells through sEVs also plays an essential role in tumorigenesis. Breast CAFs-derived sEVs express a high level of ADAM metallopeptidase domain 10 (ADAM10), enhancing cell motility by activating RhoA signaling in BC cells [138].

CAFs-derived sEVs are also crucial in cancer cell metabolic reprogramming, which is a hallmark of cancer development. CAFs-derived sEVs from prostate cancer, pancreatic cancer and BC inhibit cancer cell mitochondrial functions by reducing the oxygen consumption rate [139,140]. In BC, CAFs-derived small nucleolar RNA host gene 3 (SNHG3), a long non-coding RNA (lncRNA) that functions as a molecular sponge of miR-330-5p to upregulate pyruvate kinase M1/M2 (PKM) expression in sEVs, has a potential role in the inhibition of mitochondrial oxidative phosphorylation and the promotion of breast tumor cell proliferation [139]. Recent evidence indicates that CD63^+^ CAFs-derived sEVs play an important role in tamoxifen resistance. miR-22 is highly expressed in CD63^+^ CAFs-derived sEVs and can target estrogen receptor 1 (ESR1) and phosphatase and tensin homolog (PTEN), while suppressing the expression of ESR1 and PTEN [134]. Loss of PTEN promotes tamoxifen resistance in BC [136,141]. Breast CAFs-derived sEVs also inhibit anti-tumor immunity. The uptake of CAFs-derived sEVs increases PD-L1 expression in BC cells [142]. In particular, increased miR-92 levels have been reported in CAFs-derived sEVs [142]. Following absorption by cancer cells, miR-92 targets large tumor suppressor kinase 2 (LATS2), which interacts with YAP1 and promotes the nuclear translocation of YAP1 in BC cells. YAP1 subsequently binds to the enhancer region of PD-L1 and stimulates transcription activity, increasing PD-L1 levels in cancer cells [142,143].

In tumor-bearing mice, CD90^low^ adipose-derived mesenchymal stem cells (ADSCs) and ADSCs-derived sEVs markedly suppressed tumor growth. In another study, antioncogenic miRNA-16-5p-loaded CD90^low^ ADSCs-derived sEVs enhanced antitumor activity in preclinical BC treatments [144]. Sun et al. detected PD-L1 in exosomes secreted from bone marrow-derived cells (BMDCs) in tumor-bearing mice, but not in healthy mice. PD-L1 expressed on the surface of these exosomes suppresses CD8^+^ T cell growth and activation both in vitro and in vivo. The transfer of exogenous exosome transfer from BMDCs promotes tumor growth, whereas the inhibition of endogenous production by BMDCs suppresses the growth of tumor [145]. Moreover, when treated with DC-derived sEVs, tumor cells induce tumor-sensitized T-cells to secrete higher levels of IFN-γ [146]. In addition, NK cell-derived sEVs loaded with BCL-2 siRNA had a promising killing potential against ER^+^ BC through the induction of annexin V, caspase 3/7, and caspase 9 [147]. 

### 3.5. sEVs Modulate BC Drug Resistance

Chemotherapy is an important strategy for the treatment of cancers; however, some patients may develop resistance to chemotherapeutic drugs. Cancer cells escape from chemotherapy-induced cell death through a variety of mechanisms. Chemoresistance mechanisms include drug efflux and inactivation, activation of pro-survival bypass signaling pathways, enhanced DNA damage repair, and induction of EMT and stem cell characteristics [148,149,150,151]. 

A recent study demonstrated that tumor-derived sEVs contribute to drug resistance via the intercellular transfer of functional resistance proteins [152]. In this regard, chemotherapies, such as paclitaxel, have been shown to influence the amount and content of tumor-derived sEVs. It has been reported that tumor cells exposed to chemotherapy shed more sEVs [153,154]. sEVs are known to mediate at least three pathways that promote chemotherapeutic drug efflux. First, sEVs mediate direct drug efflux. BC cells encapsulate the chemotherapy drug doxorubicin (DOX) into vesicles, which are simply secreted out of the cells [155]. Second, sEVs promote the expression and function of membrane-embedded drug efflux pump in susceptible cancer cells. The ATP-binding cassette (ABC) transporter uses ATP to excrete a wide range of exogenous substances, including anticancer drugs, to different degrees [156,157]. The ABC subfamily B member 1 gene encodes the drug transporter permeable glycoprotein P-gp (permeability glycoprotein) [158]. Drug-resistant cancer cells have been shown to transfer P-gp proteins to sensitive cells via sEVs, resulting in the transformation of sensitive cells to resistant phenotypes [159,160,161]. Lv et al. reported that docetaxel (DOC)-resistant BC cells transported the P-gp proteins into DOC-sensitive cells via sEVs, resulting in the transfer of DOC-resistance [162]. Third, sEVs modulate the expression of metastatic functional proteins/miRNAs that regulate P-gp protein. Transient receptor potential channels (TRPC) are involved in the upregulation of P-gp proteins in resistant BC cells [163]. TRPC is involved in the sEV-mediated Adriamycin (ADM)-resistance in MCF7, a BC cell line. Internalization of TRPC5-containing sEVs induces Ca^2+^ influx in drug-sensitive MCF7 cells via TRPC5 channels, increasing P-gp proteins expression [164,165]. miR-155 was accumulated in sEVs isolated from stem-like BC cells with chemoresistance. Moreover, a line of evidence indicates the horizontal transfer of miR-155 from chemoresistant cells to recipient-sensitive cells through sEVs cargo [166]. ER^+^ breast tumors have been shown to transform from an endocrine-sensitive/dormant state to a resistant state by acquiring host mitochondrial DNA; this promotes oxidative phosphorylation (OXPHOS) and signals involved in the transition from metabolic quiescence to the state of hormonal therapy resistance [167].

sEVs, on the other hand, promote drug inactivation by transferring drug-metabolizing enzymes. GSTP1 is a drug metabolic enzyme that catalyzes phase II metabolism. It binds to glutathione and detoxify a wide range of anticancer drugs [168]. Yang et al. demonstrated increased expression of GSTP1 in ADM-resistant cells, which actively secreted sEVs. Furthermore, ADM-resistant cell-derived sEVs endow sensitive cells with a drug-resistant phenotype [76]. Evidence shows that drug-resistant tumor cells gain chemoresistance by encasing chemotherapeutic drugs into sEVs and excreting them [169,170]. sEVs contain a large amount of genetic material and are exchanged among cells in TME. Drug-resistant tumor cells act on sensitive cells through sEVs, conferring resistance [171]. Binenbaum et al. demonstrated that miR-365 in macrophage-derived sEVs transfer gemcitabine resistance into pancreatic cancer cells both in vitro and in vivo [172]. It has been shown that sEVs transferred miR-155 into MCF-7 and MDA-MB-231 BC cells, where it upregulates EMT marker molecules, targets mRNAs of TGF-β and FOXO-3a and confers C/EBP-β-induced BC resistance [166]. Inhibitors targeting anti-apoptotic pathways have been demonstrated to improve tumor cell chemosensitivity, because acquired or intrinsic chemotherapeutic resistance is frequently attributed to anti-apoptotic mechanisms in tumor cells, which lead to poor patient outcomes [173]. LncRNA-SNHG14 promotes trastuzumab resistance in HER2^+^ BC by altering the BCL-2/BAX signaling pathway; in addition, drug-resistant cells transfer lncRNA-SNHG14 to sensitive cells via sEVs, hence propagating trastuzumab resistance [174].

Tumor-derived sEVs regulate TME by suppressing immune cell responses and activating immunosuppressive cells, which is a novel mechanism of tumor resistance [175]. Neutralizing antibody drugs via tumor-derived sEVs is another mechanism for reducing anti-tumor therapeutic efficacy [176]. It was demonstrated that BC cell lines highly expressing HER2 secreted sEVs containing high levels of HER2 molecules following the treatment with trastuzumab (an anti-HER2 antibody). Therefore, trastuzumab possibly interacts with HER2-enriched sEVs in TME, reducing its therapeutic effects against primary tumor cells [177]. Furthermore, HER2^+^ BC cells develop trastuzumab resistance by secreting sEVs that contain the immunosuppressive cytokine TGF-β1 and the lymphocyte activation inhibitor PD-L1 [178].

## 4. sEV-Based BC Diagnosis and Therapy

sEVs attract much attention as cancer biomarkers; there are a wide range of substances, including proteins, DNAs, mRNAs, miRNAs, lncRNAs, and circRNAs in sEVs. Some of the substances could be utilized as biomarkers for early cancer detection, diagnosis, prognosis prediction and therapeutic efficacy evaluation [179] (Table 2). As nanoscale vesicles, sEVs offer a superior ability to traverse tissue barriers, such as the blood-brain barrier, and are prevalent in a wide range of bodily fluids, making them accessible and detectable [180,181]. 

Several studies have focused on sEV-derived non-coding RNAs. Some miRNAs in sEVs have been used as biomarkers for BC diagnosis. Hannafon et al. detected high levels of miR-21 and miR-1246 in BC patients [123]. Shimomur et al. examined serum miRNA profiles using a highly sensitive microarray system, identifying a combination of five miRNA (miR-1246, miR-1307-3p, miR-4634, miR-6861-5p and miR-6875-5p) capable of detecting BC with high sensitivity, specificity, and accuracy, even in the case of ductal carcinoma in situ (DCIS) [182]. In another study, Fu et al. found high levels of miR-382-3p and miR-1246 in the sera of BC patients, while miR-598-3p and miR-184 levels were significantly low [183].

Proteins expressed on the surface as well as the inside of sEVs could be exploited as cancer biomarkers. Tetraspanins, as previously stated, are abundantly expressed in sEVs [184]. These proteins belong to a protein superfamily that interacts with many different transmembrane and cytosolic signaling proteins [185,186]. Tetraspanin CD9, metalloprotease ADAM10, heat-shock protein HSP70, and annexin-1, in particular, are common marker proteins detected in sera and pleural effusion-derived sEVs from bodily fluids of BC patients and culture supernatants of BC cell lines [187]. Wang et al. recently showed that the level of tetraspanin CD82 in sEVs was significantly higher in the sera of BC patients than that of healthy controls and that the CD82 expression level was closely correlated with malignant BC progression [188]. Furthermore, the combination of tetraspanin CD63 and miR-21 expression in urinary sEVs yielded a 95% sensitivity in early BC detection, despite the fact that neither marker is exclusive to BC [189]. According to Rupp et al., the epithelial cell adhesion molecules EpCAM and CD24 were selectively detected in cancer-derived sEVs in ascites and pleural effusions of BC and ovarian cancer patients [190]. Moon et al. also reported that plasma levels of circulating sEVs containing developmental endothelial locus-1 protein (Del-1) and fibronectin were significantly higher in BC patients than in controls [191,192]. However, levels of the sEVs returned to normal once the tumor was removed, indicating that they were correlated with the number of tumor cells. Khan et al. demonstrated that sEVs-survivin, particularly survivin-2B, could be used as a diagnostic and/or prognostic marker in early BC patients [193]. HER2^+^ sEVs modulate Trastuzumab sensitivity and, subsequently, HER2-driven tumor aggressiveness in HER2-overexpressing BC cell lines [177]. Although not specific to early BC diagnosis, HER2 could be a useful biomarker for predicting drug resistance during treatments, which is the primary limiting factor in the development of cancer therapies. Melo et al. also detected glypican-1 (GPC1), a cell surface proteoglycan, that is selectively concentrated on cancer cell-derived sEVs [194]. They reported that GPC1^+^ circulating sEVs were detected in sera of pancreatic cancer patients with high sensitivity and selectivity. Elevated GPC1 levels have been also reported in sEVs from BC cells, implying that this sEVs biomarker could be used for early BC detection [195]. Chaudhary et al. demonstrated higher expression of serum sEVs-annexin A2 (exo-AnxA2) in BC patients than non-cancerous females, particularly for TNBC, rather than luminal and HER2^+^ BC [196]. Besides, elevated exo-AnxA2 expression in BC was closely associated with tumor grade, poor overall survival, and poor disease-free survival [196]. Another study demonstrated that exo-AnxA2 promoted angiogenesis [84]. These results strongly suggest that exo-AnxA2 is a potential prognostic biomarker and therapeutic target for TNBC [84,196].

**Table 2:** sEVs’ proteins/RNAs of breast cancer possibly used for diagnostic/prognostic biomarkers for breast cancer.

Contents in sEVs	Biomarkers in sEVs	Functions (Mechanism)/Usage	Ref.
Protein	ADAM10, Annexin-1, CD9, metalloprotease, and HSP70	Activation of RhoA and Notch signaling. Promotion of cell motility and tumor progression.	[138,187]
	CD82	Negative correlation between CD82 expression in tissues and CD82 content in exosomes.	[188]
	GPC1	Overexpression of GPC1-mediated epithelial–mesenchymal transition (EMT), which promotes invasion and migration.	[195]
	Annexin A2	Serum exo-AnxA2 is high in AA women with TNBC and promotes angiogenesis.	[84,196]
	Developmental endothelial Locus-1 (Del-1)	Plasma Del-1 levels are significantly high (*p* < 0.0001) in patients with breast cancer, but return to normal after tumor removal.	[191]
	Survivin-2B	The protein may be used as a biomarker for patients with early breast cancer.	[193]
miRNA	miR-21, miR-1246	Circulating exosomal miRNAs, miRNA-21 and miRNA-1246, are abundant in patients with breast cancer.	[123]
	miR-181d-5p	Targeting CDX2 and downregulation of CDX2 and HOXA5. Enhancement of breast cancer aggressiveness.	[134]
	miR-21, miR-378e, miR-143	Induction of stemness and EMT phenotype of breast cancer.	[100]
	miR-500a-5p	Targeting USP28 and downregulation of USP28. Promotion of proliferation and metastasis of breast cancer cells.	[135]
	miR-4516	Targeting FOS such as antigen 1 (FOSL1). Promotion of TNBC development.	[197]
	miR-22	Targeting ESR1 and PTEN and downregulation of ESR1 and PTEN. Promotion of tamoxifen resistance.	[136]
	miR-1246	The exosomal miRNA-1246 in the peripheral blood can distinguish 46 breast cancer patients from 28 healthy controls with 100% sensitivity and 93% specificity.	[61]
lncRNA	SNHG3	Targeting miR-330-5p and promotion of PKM expression and glycolysis metabolism.	[139]
circRNA	circHIF1A	Induction of CD44 expression by targeting and down-regulating miR-580-5p. Promotion of breast cancer cell proliferation and stemness in hypoxic stress.	[137]

sEVs have been considered as promising drug delivery agents because of their inherent intercellular communication roles, excellent biocompatibility, low immunogenicity, low toxicity, extended blood circulation ability, biodegradable characteristics and ability to traverse numerous biological barriers [198,199,200,201] (Table 3). Clinically, nanotechnology-based drug delivery systems are one of the state-of-the-art techniques for achieving this goal. sEVs have been used successfully as drug and functional RNA delivery vectors in cancer treatment due to their intrinsic delivery capabilities [202]. sEVs can be absorbed by cells and can stably convey drugs, including therapeutic miRNAs and proteins [203]. Compared to other drug carriers, such as liposome nanomaterials, metal nanomaterials and polymer nanomaterials, sEVs can overcome the disadvantages of poor bioavailability and reduce non-targeted cytotoxicity and immunogenicity [201,204]. In addition, there are numerous transmembrane and membrane anchoring proteins in sEVs, which enhance endocytosis and promote the subsequent transfer of their internal contents [205,206].

Furthermore, sEV-based delivery platforms are superior to free drugs in terms of reduced side effects [200]. For instance, doxorubicin cardiotoxicity limits chemotherapy dosages for BC patients. Toffoli et al. demonstrated that sEVs did not alter the efficacy of DOX, whereas exosomal doxorubicin decreases cardiotoxicity and adverse effects on other tissues, when compared to free doxorubicin; as such, increasing doxorubicin doses would counteract increased toxic effects on BC cells [207]. In a recent study, sEVs surface modification was performed using oligonucleotide binding techniques; such cargoes may influence not only cell functions, but also cell-to-cell transport [208]. TNBC is the most metastatic and recurrent subtype of BC. Li et al. modified the surface of the sEVs using a peptide to target, the EMT factor gene c-Met, which is a tyrosine kinase receptor for hepatocyte growth factor or scatter factor overexpressed on TNBC cell surfaces [209]. These modified sEVs increased cellular uptake efficiency and the antitumor efficacy of doxorubicin [210]. 

Rapidly advancing nanotechnologies enable the use and engineering of sEVs for therapeutic purposes, resulting in the emergence of a new class of cell-free nanomedicine. Therapeutic blockade of the exosome synthesis to limit cancer progression at specific stages of the disease could be appealing in the development of cancer therapeutics [211,212,213]. A recent study demonstrated a potential application of responsive exosome nano-bioconjugates to cancer therapy; the nano-bioconjugates could actively target tumors by specific recognition on the surface of tumor cells and abolished signaling and improved macrophage phagocytosis [214]. Engineered sEVs are gaining popularity as potential therapeutic vehicles or active drug delivery systems [29,215,216,217]. Using the organotropic characteristics of sEVs, sEVs loaded with therapeutic compounds could be employed to selectively target recipient cells for gene therapy.

Trastuzumab emtansine (T-DM1) uses an antibody-drug conjugation approach to deliver DM1, a cytotoxic drug to Her2^+^ cancer cells. Cancer-derived sEVs also contain the target of T-DM1 (Her2). It was then examined whether exosome-bound T-DM1 contributes to T-DM1 activity. As a result, T-DM1 was transferred to other cancer cells via sEVs derived from HER2^+^ cancer cells, leading to decreased viability of the recipient cells. Therefore, trastuzumab-emtansine is transported from HER2^+^ cancer cells into cancer cells through cancer-derived sEVs, resulting in growth suppression and caspase activation [218].

Both immature and mature DCs produce exosomes, and DC-derived exosomes (DEXs) can overcome tumor-induced immunosuppression in both direct and indirect ways. sEVs derived from mature DCs express higher levels of MHC I, MHC II and costimulatory molecules than those secreted from immature DCs, implying that mature DC-derived sEVs have potent immune-stimulating effects [219]. There are MHC/peptide complexes on the surface of DEXs, which induce the proliferation and activation of IL-15R*α* and NKG2D-expressing NK cells and enhance T cell-dependent antitumor activity [220]. In addition, DEXs potentially activate T cells, promote T cell-mediated immune responses, and inhibit the growth of BC cells [146]. DEXs-based anticancer vaccines have entered phase I and II clinical trials following animal model testing [221,222]. 

**Table 3:** Possible application of sEVs to breast cancer therapy.

Payload/Drug Names	Targets	Ref.
Cell cycle quiescence and chemotherapy-resistant mesenchymal stem cells-derived ssEVs	Facilitating breast cancer cells to progressively differentiate into dormancy in the perivascular region of bone marrow	[223]
Tumor-acclimated MSCs-derived ssEVs	Induction of monocytic myeloid-derived suppressor cells	[224]
M1-type macrophages-derived ssEVs loaded with Paclitaxel (PTX)	Microtubule	[225]
Human fetal lung fibroblast-derived ssEVs loaded with FA and irastatin	Ferroptosis/Folic acid	[217]
Hybrid Exosome (HE) loaded with water-soluble doxorubicin	DNA	[226]
Generalized exosomal nanobiological conjugate produced by pH-responsive macrophage M1 binding with anti-tumor effect of antibodies	Specific proteins	[214]
Exosomes isolated from MSCCXCR4 + TRAIL transduced with CXCR4 and TRAIL (exosome CXCR4 + TRAIL) loaded with carboplatin	Synthesis of DNA	[227]
Biomimetic tumor-derived exosomes loaded with (TEX)-Liposome-paclitaxel (PTX)	Microtubule	[228]
Folic acid (FA)-functionalized bovine milk-derived ssEVs loaded with Paclitaxel (PAC) and 5-fluorouracil (5-FU)	Microtubule/synthesis of DNA	[229]
miR-567	Cancer-associated ATG5	[230]
let-7a miRNA	EGFR	[231]
Ultrasonic sensitizer indocyanine green (ICG)	Folic acid	[232]
Deintegrin and metalloproteinase 15 (A15), doxorubicin (Dox) and cholesterol-modified miRNA 159	DNA/GAMYB	[233]
Cationic bovine serum albumin (CBSA) conjugated siS100A4 nanoparticles	Lung PMN	[234]
Surface display monoclonal antibodies against human CD3 and human HER2	Human CD3 and human HER2	[235]
The conjugates of reactive oxygen species (ROS)-responsive sulfide-linked paclitaxel-Linoleic acid conjugates (PTXS-LA) and cucurbitin B (CuB) (EMPC) loaded with CD44	Circulating tumor cells	[236]

## 5. Conclusions and Challenges

**Figure 2:**
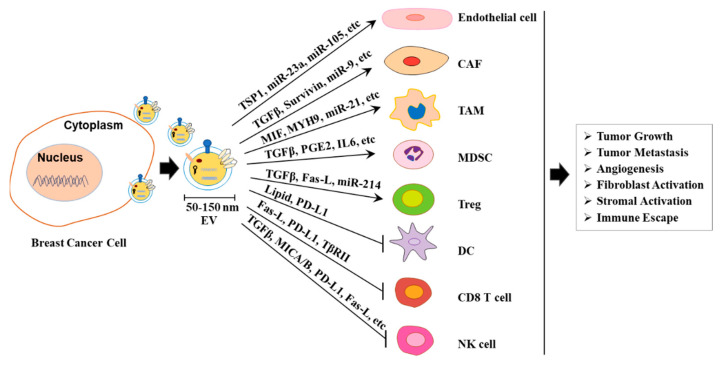
Schematic diagram illustrating functional pathways and target cells of BC-derived sEVs. CAF; cancer-associated fibroblasts; TAM: tumor-associated macrophage; MDSC: myeloid-derived suppressor cells; Treg: regulatory T cells; DC: dendritic cells; NK cell: natural killer cell.

In conclusion, BC-derived sEVs alter phenotypes and functions of endothelial cells, CAFs, and immune cells in TME through various pathways, thereby promoting tumor growth, metastasis, and immune escape (Figure 2). 

Liquid biopsies identify circulating tumor cells, sEVs, DNAs, exosomes, and microRNAs, in which sEVs attract much attention because of their critical roles in cancer regulation. The benefit of sEVs stems from their ubiquitous presence, unique DNA/RNA/protein compositions, and high efficiency in targeting target cells. However, there are several hurdles to using sEVs in preclinical studies and clinical practices. First, the development of quantitative and reproducible purification procedures is required for the preparation of preclinical and clinical samples. Second, cell culture conditions, such as cell passage, cell density, harvest frequency, and other culture parameters, have a significant impact on the quality of sEVs preparations, including yield, sEVs compositions and bioactivity. Third, the use of sEVs as a drug delivery system should address the question of whether loading exogenous cargo interacts with endogenous cargo and whether this has an off-target effect. Finally, the use of sEVs as biomarkers in the clinical setting requires numerous validations following initial discovery. Additional in-depth investigation is, therefore, required to exploit the translational potential of sEVs in the development of BC diagnosis and therapy.

## Data Availability

Not applicable.
